# Is the abundance of *Faecalibacterium prausnitzii* relevant to Crohn's disease?

**DOI:** 10.1111/j.1574-6968.2010.02057.x

**Published:** 2010-08-02

**Authors:** Wenjing Jia, Rebekah N Whitehead, Lesley Griffiths, Claire Dawson, Rosemary H Waring, David B Ramsden, John O Hunter, Jeffrey A Cole

**Affiliations:** 1 School of Biosciences, University of BirminghamBirmingham, UK; 2 Gastroenterology Research Unit, Addenbrookes HospitalCambridge, UK; 3 Medical School, University of BirminghamBirmingham, UK

**Keywords:** Crohn's disease, ulcerative colitis, irritable bowel syndrome, *Faecalibacterium prausnitzii*, butyrate, faecal bacteria

## Abstract

Reports that bacteria within the *Firmicutes* phylum, especially the species *Faecalibacterium prausnitzii*, are less abundant in Crohn's disease (CD) patients and supernatants from cultures of this bacterium are anti-inflammatory prompted the investigation of the possible correlations between the abundance of *F. prausnitzii* and the response to treatment in patients with gut diseases and healthy controls. In a randomized, double-blind trial, faeces were collected from healthy volunteers, and from patients with active CD, ulcerative colitis (UC) and irritable bowel syndrome before and after treatment. The levels of *F. prausnitzii* DNA in faecal suspensions were determined by PCR. Treatment by an elemental diet was effective, resulting in decreases in both the Harvey and Bradshaw index (*P*<0.001) and the concentrations of serum C-reactive protein (*P*<0.05). The total levels of *F. prausnitzii* in faecal samples from CD patients at presentation were lower than those in the other groups both before and after the treatment. There was no correlation between *F. prausnitzii* abundance and the severity of CD before treatment. Clinical improvement unexpectedly correlated with a significant decrease in the abundance of *F. prausnitzii*, especially the A2-165 subgroup (*P*<0.05). Our data suggest that a paucity of *F. prausnitzii* in the gastrointestinal microbial communities is likely to be a minor aetiological factor in CD: recovery following elemental diet is attributed to lower levels of gut flora.

## Introduction

The cause of Crohn's disease (CD) is unknown and successful, long-term treatment represents considerable clinical and pharmacological challenges. The conventional treatment of disease of mild to medium severity consists of antibiotics to counteract infection and 5-aminosalicylic acid (5-ASA) with or without corticosteroids to counter inflammation, but remission can also be achieved with enteral feeding (King *et al.*, 1997). The faecal bacterial flora in CD is known to be abnormal (Sartor, 2008). CD has been suggested to be an autoimmune disease with an immune attack directed against the resident bacteria of the colon (Macpherson *et al.*, 1996). The coating of colonic bacteria with immunoglobulin approaches 100% in active CD, as compared with 20% in healthy subjects and patients with irritable bowel syndrome (IBS). After enteral feeding, this coating is rapidly reduced (van der Waiij *et al.*, 2004).

There is increasing evidence that gut microorganisms play key roles in gastrointestinal disease (Sartor, 2006; 2008;). Some are believed to generate a harmful toxin (Pitcher & Cummings, 1996; Loubinoux *et al.*, 2002;). Many others are known to yield butyrate, which is not only an essential energy source for colonocytes (Chapman *et al.*, 1994; Costello *et al.*, 1994; Macpherson *et al.*, 1996; Aminov *et al.*, 2006; Louis & Flint, 2009; Mai & Draganov, 2009;) but also is both anti-inflammatory and anticarcinogenic (Hamer *et al.*, 2008; Tazoe *et al.*, 2008;). Therefore, considerable interest has been generated by reports that a reduction in bacteria within the *Firmicutes* phylum, especially the species *Faecalibacterium prausnitzii*, has been observed in the gut of CD patients and that supernatants from cultures of this bacterium are anti-inflammatory (Sokol *et al.*, 2008). Because oxidation of butyrate is the major source of ATP in cells lining the colon (Yin *et al.*, 2001), decreased levels of *F. prausnitzii* could well reduce ATP within these cells. At the same time, coupled with the loss of anti-inflammatory agents from *F. prausnitzii*, this would, in a double blow, weaken the ability of the cells to fight infection (Smith *et al.*, 2008). Thus, these findings potentially shed light on the aetiology of CD and offer a novel therapeutic approach involving recolonization of the gut with the bacterium.

To evaluate these findings and to assess the effects of elemental diet therapy (ED) on the population of *F. prausnitzii* within the gut, faecal samples were collected before and after therapy from patients suffering from CD, IBS and ulcerative colitis (UC) as disease control groups, and also from healthy controls.

## Materials and methods

### Patients and control subjects

Twenty patients diagnosed to be suffering from CD, 21 patients with IBS, 14 patients with UC and 18 healthy subjects as controls were recruited at Addenbrookes Hospital, Cambridge, which functions as a tertiary referral centre for inflammatory bowel disease. A faecal sample and a blood sample were taken at the start of the treatment and again (except for the controls) 2 weeks later. Faecal samples were collected at the homes of patients and control subjects and immediately stored at 4 °C. Samples were taken to Addenbrookes Hospital, assigned undisclosed identification codes and sent by courier at 4 °C to the University of Birmingham for DNA extraction, arriving within 48 h of collection. Sample identifications were decoded only after data collection had been completed. Ethical permission to collect these samples was obtained from Leeds West Ethics Research Committee, ref. 07/Q1205/39, and informed, written consent was obtained from each subject.

### Treatment regimes and assessment of the effects of treatment

Most CD patients attending the Addenbrookes Hospital tertiary referral clinic were considered to be relatively complex cases that had proved refractory to previous pharmacological treatments. These included steroids and/or immuno modulation therapy (azathioprine or methotrexate), or 5-ASA. Treatment was chosen by the individual consultant supervising each case, but all included in the present study were given ED (King *et al.*, 1997). All normal food items were withdrawn and patients were maintained for 2 weeks on ED (E 028 Extra, Scientific Hospital Supplies International, Liverpool, UK), which contains a predigested mixture of amino acids, malto-dextrins, minerals and vitamins, with a single fat source, rape seed oil. Thus, no additional complex carbohydrate or protein was provided for the gut flora to use as energy substrates other than endogenous sources, for example, protein released from sloughed cells. Water *ad libitum* was the only other item allowed. The nutritional requirements were individually calculated using the Schofield equation (Schofield, 1985). The efficacy of treatment was assessed on symptoms using the Harvey and Bradshaw index (Harvey & Bradshaw, 1980), and by an assay of serum C-reactive protein (CRP) as a measure of inflammatory stress.

None of the UC and IBS patients was given ED. Eleven of the UC patients had received either immuno-modulation or 5-ASA therapy before this study, and this was continued, albeit with changes in the drugs and increases in the doses used. Treatment-naïve patients were placed on similar regimens. IBS patients received conventional treatment.

The Harvey and Bradshaw index and the Walmsley index were used to determine the severity of CD and UC, respectively.

### DNA extraction

DNA was extracted from each faecal sample (0.2 g) using a QIAamp DNA stool Mini Kit (Qiagen) by following the manufacturer's instructions. Extracted DNA was stored in aliquots at −20 °C. DNA quality was assessed by determining the ratio of A_260 nm_ to A_280 nm_.

### PCR amplification of bacterial DNA

Two subgroups of *F. prausnitzii* have been identified on the basis of their published whole-genome DNA sequences. Primers Fp.ID.F2 (GTGACCGGATCGAACGACC) and Fp.ID.R2 (TCCAGGTCATGTGGGCAGC) were designed against the nucleotidyl transferase gene and the butyryl-CoA transferase gene, respectively, using the nucleotide sequences of both *F. prausnitzii* A2-165 and M21/2. Amplicons from the two *F. prausnitzii* subgroups could be generated simultaneously within a single PCR reaction and distinguished by the fragment length: approximately 650 bp for the A2-165-related subgroup and 778 bp for the M21/2-related subgroup. For each PCR amplification, 50 ng of DNA was used as a template with HotStarTaq Plus Master Mix Kit (Qiagen) plus 4 μL of Q-solution (Qiagen) according to the manufacturer's instructions. The reaction conditions were: initial denaturation (5 min at 95 °C), then 35 cycles of denaturation (1 min at 94 °C), annealing (1 min at 58 °C) and elongation (1 min at 72 °C) and a final extension (10 min at 72 °C).

For samples from which *F. prausnitzii* was not amplified, the quality of the DNA was confirmed using 50 ng of DNA as template in a second PCR with the primers 27F (5′-AGAGTTTGATCATGGCTCAG-3′) and 1492R (5′-GGTTACCTTGTTACGACTT-3′) that annealed to bacterial DNA encoding 16S rRNA gene (Lane, 1991). These primers amplify a highly conserved region in the DNA from many bacterial species in human faeces. The reaction conditions were as follows: initial denaturation (5 min at 95 °C), then 35 cycles of denaturation (30 s at 94 °C), annealing (1 min at 55 °C) and elongation (2 min at 72 °C) and a final extension (8 min at 72 °C). The reaction mixture was the same as stated above.

### Quantification of amplified DNA

Because the same amount of template DNA, 50 ng, was added to each PCR, the differing brightness of the bands on the gel was used as an indicator of the amount of *F. prausnitzii* in each patient. The intensity of the bands was measured using the software quantity one (Bio-Rad) and quantified by comparison with known amounts of a 1-kb ladder (New England Biolabs). The experiment was repeated once. Samples that failed to yield a PCR product were reassayed a third or a fourth time, and the quality of the DNA was confirmed by amplification of bacterial 16S rRNA gene.

### Serum CRP assay

Serum CRP levels were determined by the Clinical Biochemistry Department, Addenbrookes Hospital.

### Statistical analyses

The results were assessed using nonparametric methods. The results of the estimated amount of each subgroup of *F. prausnitzii* DNA generated by PCR amplification for the three patient groups both pre- and post-treatment and the control group were analysed separately using Kruskal–Wallace one-way anova and Dunn's *post hoc* all comparisons test. To gain an estimate of the effects of illness on the total amount of *F. prausnitzii*, the results for the controls and the three pretreatment samples were ranked first for the M21/2 subgroup (the larger PCR fragment) and then for the A2-165 subgroup (the smaller PCR product), and the two ranking values for each sample were added together to yield a combined rank value that was regarded as an estimate of the total *F. prausnitzii* content of the faeces. Post-treatment samples were analysed in the same way. These two new data sets were also analysed using the Kruskal–Wallace one-way anova and Dunn's *post hoc* all comparisons. Paired data were analysed using the Wilcoxon signed rank-sum (WSRS) test. Kruskal–Wallace analyses were carried out initially using the minitabs statistical package. Data were then reanalysed using the instat (Graphpad) program, which was also used for Dunn's *post hoc* all comparisons (but completed only if the two-tailed *P* value was <0.05 in the Kruskal–Wallace analysis), Mann–Whitney tests for unpaired data, the WSRS test for paired data and Spearman *r* to determine the level of correlation between parameters.

## Results

The results are summarized in Tables 1 and 2. The original data on which these tables are based are available as Supporting Information, Tables S1–S3 in the online version of this article.

**Table 2:** Kruskal–Wallis test for data on the abundance of the *Faecalibacterium prausnitzii* subgroup in different groups of patients

		A2-165 subgroup			M21/2 subgroup		
Group	*N*	Median	Average rank	*Z* value	Median	Average rank	*Z* value
Control vs. before treatment	18	178	39.3	0.52	275	43.9	1.60
Control vs. after treatment	18	178	42.8	1.32	275	45.3	1.90
CD before treatment	20	77	29.1	−1.95	77	28.3	−2.16
CD after treatment	20	0	23.4	−3.36	66	26.5	−2.61
UC before treatment	14	177	38.4	0.28	110	30.2	−1.33
UC after treatment	14	141	38.4	0.27	154	34.9	−0.42
IBS before treatment	21	180	41.6	1.18	241	43.9	1.76
IBS after treatment	21	192	44.1	1.32	197	41.4	1.13

**Table 1:** Yields of PCR products related to the two *Faecalibacterium prausnitzii* subgroups from the different patient groups

		A2-165 subgroup		M21/2 subgroup	
Group	*N*	Mean	SD	Mean	SD
Control	18	170	135	248	161
CD before treatment	20	103	103	127	144
CD after treatment	20	44	74	113	138
UC before treatment	14	170	147	149	164
UC after treatment	14	157	162	175	164
IBS before treatment	21	202	162	246	149
IBS after treatment	21	180	131	214	111

*N*, number of samples; Means, average yield (ng) of PCR product generated; SD, SD within the sample.

### Subject characteristics

The patient groups consisted of 20 patients diagnosed as suffering from CD, 21 patients with IBS and 14 patients with UC. They were recruited at Addenbrookes Hospital, Cambridge, which functions as a tertiary referral centre for inflammatory bowel disease. The patients gave a faecal and a blood sample at the start of the treatment and again 2 weeks later. In addition, blood and faecal samples were obtained from 18 healthy subjects as controls.

### Effects of treatment on the clinical state

Eighty-four per cent of the initial group of CD patients returned to follow-up clinics. The effects of treatment were beneficial, as evidenced by decreases in the Harvey and Bradshaw scores of individual patients (*P*<0.0001; WSRS test). At presentation, all 15 patients whose serum was assayed had serum CRP levels above the upper limit of the reference range (6 mg L^−1^). Following treatment, there was a significant decline in the CRP levels (*P*<0.05, WSRS test).

### Quantification of *F. prausnitzii*-specific PCR products

**Fig. 1:**
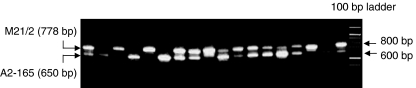
Relative quantity of *Faecalibacterium prausnitzii* in faecal DNA determined by PCR. Agarose gel showing bands of the *F. prausnitzii* amplicon from 22 faecal samples. The upper band of 778 bp corresponds to the M21/2 subgroup; the lower 650 bp band corresponds to the A2-165 subgroup. The same amount of template DNA was added to each PCR reaction, and so these differences indicate the relative amount of *F. prausnitzii* in each patient. Where a PCR product was present, the brightness of the band was measured using the software quantity one (Bio-Rad) and converted to DNA amount by reference to a 1 kb ladder (New England Biolabs: right-hand lane).

Bacterial DNA was obtained from each faecal sample. The same amount of faecal DNA was used as a template in PCR reactions with the primer pair Fp.ID.F2 and Fp.ID.R2 to detect the presence of *F. prausnitzii*. Although the total quantity of *F. prausnitzii* PCR products and the ratio of the two subgroups varied considerably from sample to sample (Fig. 1), only a minority of samples failed to yield one or both *F. prausnitzii* PCR products. The sequences of PCR products were 100% identical to those of the published *F. prausnitzii* genomes A2-165 or M21/2, and to no other. When no *F. prausnitzii* product was detectable, a second amplification to produce an amplicon from the DNA encoding the 16S rRNA gene was always successful, demonstrating that the bacterial DNA extracted was of good quality.

### Comparison of *F. prausnitzii* abundance in the faecal samples

There were no significant differences in the levels of the A2-165 subgroup of *F. prausnitzii* in the three treatment groups at initial presentation and in the control group (Kruskal–Wallace *P*=0.248, adjusted for ties), although the levels for CD patients were the lowest of the four groups. There was a significant difference among the three post-treatment and control groups (Kruskal–Wallace *P*=0.006, adjusted for ties), with the levels for the CD group being significantly lower than those of the control group (Dunn's *post hoc* test *P*<0.05). For the CD group, the levels had decreased significantly post-treatment (WSRS test *P*=0.0046).

The levels of the M21/2 *F. prausnitzii* subgroup were analysed similarly. There was a significant difference among the three pretreatment patient groups and the control group (Kruskal–Wallace *P*=0.029, adjusted for ties), although the *post hoc* test revealed no significant difference when individual groups were compared with each other, despite lower values for both the CD and the UC samples. However, for this *F. prausnitzii* subgroup, the levels in the CD patients were significantly lower than those in the control samples, as judged by the Mann–Whitney test (*P*=0.02). Following treatment, there was also a significant difference in the levels of the M21/2 subgroup among the three patient groups and the control group (Kruskal–Wallace *P*=0.034, adjusted for ties). The levels in the CD group were significantly lower than those in controls (Dunn's *post hoc P*<0.05), although the difference between the pre- and the post-treatment levels (median before treatment 77 compared with 65.5 post-treatment) was not significantly different (WSRS *P*=0.61). Thus, the levels of both *F. prausnitzii* subgroups for CD patients either decreased or remained low rather than increased following successful treatment with an enteral diet.

It is obvious from Fig. 1 that there were wide differences between the ratios of the levels of the two *F. prausnitzii* subgroups between patient samples. However, the overall total levels of each subgroup were similar, and so it was appropriate to lend equal weight to the two subgroups to compare the total levels of *F. prausnitzii* among the patient and control populations. The analyses revealed that the total levels of *F. prausnitzii* in CD patients were significantly lower than those in the other groups both before and after treatment (Kruskal–Wallace test *P*<0.005 before treatment and <0.001 after treatment; Dunn's comparisons for the CD group relative to the control group *P*<0.05 before treatment; <0.001 after treatment).

### Lack of a correlation between the levels of *F. prausnitzii* and clinical assessment

The levels of each subgroup of *F. prausnitzii* in CD patients were analysed for correlation with clinical status, as determined by the Harvey and Bradshaw scores, both before and after treatment. The data were assessed using the nonparametric Spearman *r* coefficient. As the *r* value for the A2–165 subgroup was 0.018 (*P*=0.3) and *r*=0.02 (*P*=0.92) for the M2/21 group, neither set of *F. prausnitzii* levels correlated with clinical status.

### Further analysis of data from samples from the UC patients

The data for UC patients before treatment appeared to show that the levels of the *F. prausnitzii* M2/21 subgroup were lower those than in the control group, but the statistical tests used failed to establish that the difference was significant. We therefore used the nonparametric Mann–Whitney test to compare the data for this UC subgroup with the controls. As the resulting *P* value, 0.087, tended towards, but did not confirm that the difference was significant, this is probably a type 2 error: more data are required for this patient group.

## Discussion

Previous studies have established that *Firmicutes* are dominant among the diverse range of butyrate-producing bacteria in the human gut, with five species being especially abundant: *Eubacterium rectal; Roseburia faecis; Eubacterium hallii*; an unnamed but cultured species, SS2/1; and *F. prausnitzii* (Aminov *et al.*, 2006; Louis *et al.*, 2009;). The report that the levels of *F. prausnitzii* in the gut of patients with CD were lower than the levels in healthy subjects was based on analysis of tissue taken from patients undergoing elective surgery (Sokol *et al.*, 2008). The current study used an alternative way of identifying bacteria present in the human gut, which was to investigate the bacterial component of faecal samples. However, analysis of faecal DNA in the present study only partially confirmed these earlier findings. Although *F. prausnitzii*, especially the M21/2 subgroup, were less abundant in CD patients at presentation than in the control group, contrary to expectation, successful treatment of the CD group resulted in a decrease rather than an increase in *F. prausnitzii* abundance. It is an open question as to whether the treatment received by CD patients before attendance at the Addenbrookes clinic might have resulted in the statistically significant decline in the faecal levels of *F. prausnitzii*: a recent study strongly suggests that this might be the case (Benus *et al.*, 2010). However, the subsequent decline following ED treatment clearly excludes the possibility that recovery might be due to an increase in *F. prausnitzii* levels within the gut and the consequent increase in immuno-suppressive agents secreted by the bacteria (Sokol *et al.*, 2008).

The causes of CD and other gastrointestinal diseases are clearly multifactorial, and it is therefore not surprising that the same is true of mechanisms that protect against disease. The previous report clearly established that the positive effect of *F. prausnitzii* in deterring the recurrence of CD following surgery was due to the production of an unidentified metabolite other than butyrate (Sokol *et al.*, 2008). Bacteria generate butyrate during fermentation of lactate, but lactate is also the preferred energy source of many other bacteria, including propionibacteria, acetogens and sulphate-reducing bacteria (Macpherson *et al.*, 1996). In the current study, patients were treated nutritionally with a minimal diet that would decrease the availability of lactate and hence also decrease the bacterial flora, including *F. prausnitzii* (Chapman *et al.*, 1994; Costello *et al.*, 1994; King *et al.*, 1997; Whelan *et al.*, 2005; Johnson *et al.*, 2006;). It is known that faecal bacterial dry weight in healthy volunteers fed ED declines drastically (Costello *et al.*, 1994). A positive outcome following treatment might therefore be due to a greater reduction in the production of toxic, inflammatory metabolites by other bacteria relative to any negative effects of a decreased population of butyrate-producing bacteria. For example, decreased availability of lactate would select against sulphate-reducing bacteria such as *Desulfovibrio piger*, which is abundant in human faeces and the major source of sulphide, which inhibits butyrate oxidation by colonocytes (Pitcher & Cummings, 1996; Roediger *et al.*, 1997; Willis *et al.*, 1997; Levine *et al.*, 1998; Loubinoux *et al.*, 2002; Duncan *et al.*, 2004; Fite *et al.*, 2004; Marquet *et al.*, 2009;).

The different results reported in this and the previous study may have arisen from the differences in the material assayed. Normally produced stools, which were used in this study, represent bacterial populations chiefly along the colon, whereas the surgically resected material in the earlier study were from higher up the alimentary canal. Bacterial populations in the two sites may well differ. Furthermore, patients awaiting surgery may have been given enteral or parenteral feeds to improve their state of nutrition or fasted preoperatively. If the organism is dependent on dietary complex carbohydrate, these also could have been factors leading to the low numbers of *F. prausnitzii* reported.

In conclusion, the clinical improvement in our patients with CD was associated with a decline in the numbers of *F. prausnitzii*, but this does not mean that the organism can be considered to be a pathogen responsible for producing CD, as it was equally frequent in our healthy volunteers. It is more likely to be an innocent bystander, whose numbers decline with ED feeding, which exerts its effects in CD through its action on the gut microbial communities (Hunter, 1991). We therefore attribute the decrease in the faecal *F. prausnitzii* levels in CD patients following ED to the fact that this diet reduces the undigested food residues required to sustain normal levels of flora in the lower gut, particularly bacteria such as *F. prausnitzii*. Such a change in flora was associated with the marked improvement seen in patients' symptoms and the levels of CRP. We suggest, therefore, that this reduction in gut flora was chiefly responsible for the patients' improvement.

## Statement

Re-use of this article is permitted in accordance with the Terms and Conditions set out at http://www3.interscience.wiley.com/authorresources/onlineopen.html

## SupportingInformation

Table S1. Quantified yields of the two PCR products from the different groups of *Faecalibacterium prausnitzii* strains in individual patient samples.

Table S2. Clinical assessment of the patient group with Crohn's disease.

Table S3. Clinical assessment of the patient group with ulcerative colitis.
